# Chinese National Air Protection Policy Development: A Policy Network Theory Analysis

**DOI:** 10.3390/ijerph15102257

**Published:** 2018-10-15

**Authors:** Xiao Gong, Jianing Mi, Ruitao Yang, Rui Sun

**Affiliations:** 1 School of Management, Harbin Institute of Technology, Harbin 150080, China; mijianing@hit.edu.cn (J.M.); sunrui@stu.hit.edu.cn (R.S.); 2 Center of Ultra-Precision Optoelectronic Instrument Engineering, Harbin Institute of Technology, Harbin 150080, China; ruitao.yang@hotmail.com

**Keywords:** air protection policy, environmental policy, policy network, Chinese government, international agreements

## Abstract

Given its wide involvement in and recognition by international organizations, China has signed many international agreements and negotiations. This study verified how and the extent to which changes in exogenous factors (e.g., international agreements and negotiations) affect Chinese governmental air protection policy development. Previous studies on policy network theory have demonstrated that exogenous factors affected the development of domestic policies significantly, while in this study little evidence was found to demonstrate the influence of exogenous factors on changes in Chinese policy. Rather, internal factors have played an important role in both its development and transformation. These findings differ from study results on wealthy countries and other developing districts. This study then explores the causes of substandard policy outcomes. To probe this further, policy network theory is applied to explain the gap between the guiding principle of central government’s policies and local implementation in actual practice. By analyzing the strategies of policy actors and specific rules in current Chinese context, the associated limitations and obstacles in the process of policy-making and implementation can be explained from the aspect of bureaucratic system, energy market running mechanism and others. This paper recommends alterations in the current policy and structure based on these findings.

## 1. Introduction

**Figure 1:**
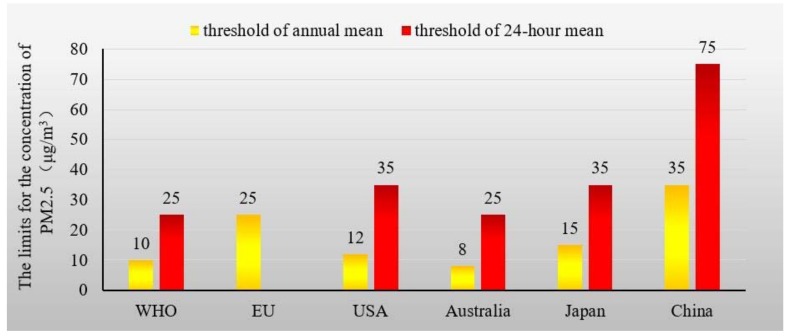
International limits for fine inhaled particulate matter with a diameter of 2.5 micrometers (PM_2.5_, μg/m^3^) concentration. (Source: Reports from IEACCC (International Energy Agency Clean Coal Centre) and WHO (World Health Organization) [9,18]).

In China, persistent and widespread haze events resulting from atmospheric particulate matter have been disturbing residents’ quality of life. These events have severely impacted public health and national economic progress [1]. Haze-fog with poor visibility has caused not only traffic problems, but also the rising of morbidity and mortality. According to an ongoing research from the American Cancer Society, people exposed to more pollutants are more susceptible to lung cancer [2]. The mortality relative risk is up to 1.14 for a 10 μg/m^3^ increase of PM_2.5_ (i.e., fine inhaled particulate matter with a diameter of 2.5 micrometers) [2]. The visibility and haze have been demonstrated to affect the mortality in a coastal city of China quite obviously [3]. Previous researches show that haze occurred just in January 2013 has caused almost 700 premature death, 45 thousand of acute bronchitis and 24 thousand of asthma cases in Beijing with a 95% confidence interval [4,5,6]. Thus, children have also been advised to stay indoors to decrease their exposure to pollutants [7]. Besides, air pollution also caused huge economic loss in the field of building, agricultural production, and social total welfare [8]. It is therefore necessary to determine an effective way to decrease air pollution. The World Health Organization (WHO) cautions against average daily exposures of PM_2.5_ at a level higher than 25 μg/m^3^ [9]. However, no more than 1% of China’s 500 largest cities meet this standard [10]. According to a monthly report on air quality involving 74 major Chinese cities in November 2015, 10 cities were “heavily polluted” [11]. In Beijing, the maximum value of daily average PM_2.5_ concentrations reached 464 μg/m^3^. In some northeastern cities (e.g., Shenyang and Harbin), the peak concentrations of PM_2.5_ have exceeded the limit, reaching 1000 μg/m^3^ [11]. It is clear that the concentrations of PM_2.5_ in Chinese cities far exceed the upper limits according to all international standards (Figure 1).

Similar to other developing countries, China has been forced to make trade-offs between its energy supply stability, pollution reduction measures, and economic development [12,13]. China has also signed international agreements (e.g., the Kyoto Protocol) established to force member countries to reduce waste-gas emissions. As an Appendix II country of the United Nations Framework Convention in Climate Change (UNFCCC), China has made a commitment to increase its 2005 share of non-fossil fuels used in primary energy consumption to approximately 15% by 2020 [14]; this may decrease the generation of fine particulate matter (PM) caused by fossil fuel combustion. Indeed, the Chinese government has issued a series of relevant policies and policy claims. Chinese municipalities also hope to create positive reputations and attractive city labels (e.g., “eco city” and “natural oxygen bar”) to attract investors and secure support from higher government tiers [15,16,17].

In academic circles, the air pollution has drawn great attention as well. A large part of these studies derive from scientific disciplines, e.g. atmospheric chemistry or meteorology, and focus on the pollutant itself [19,20,21,22,23]. In the field of social science, many researches are empirical analysis and study the loss in monetary value primarily [24,25]. Public health is also an important research subject [26,27,28,29]. The existing analysis on relevant policies are either broad from a national perspective, or just narrow down to a certain type of policy. As a breakthrough, Jin made a complete and detailed retrospective on Chinese air protection policies. The different policy changes and the drivers of these changes are analyzed [30]. Key factors influencing the effectiveness of policies are illustrated in this paper as profound changes in social development and redistribution of power as well.

This paper aims to identify what factor directly influences the development of Chinese air protection policies. It needs to be clear whether the exogenous factor is also the key factor, just as the previous studies on other countries and economies. The paper further reports an explanation of the gap between the guiding principle of central government’s policy and local implementation in actual practice. The method for approaching these questions are not from atmospheric chemistry, economics or policies’ history perspective like the studies mentioned above. Instead, a more distinct governance perspective centering policy implementation through policy network is applied in this paper. Although implementation has been a classical topic in public policy since Pressman explored the reasons for the failure of Washington DC’s ambitiousness on policy in Oakland [31], it is still overlooked frequently when it comes to substandard policy outcomes [32]. Because the policy in China is implemented in a top-down approach, policy outcome relies on a rational bureaucratic system rather than the interrelationships among actors in policy network [33,34]. The underemphasizing of interrelationships among actors leads to the predictable substandard policy outcome in actual process of implementation. Therefore, the interdependence among the actors should be given more attention and policy network is essential in this process. This paper first introduces the policy network theory and then examines the effects of exogenous factors on Chinese air protection policy changes, including international agreements and negotiations. The history of Chinese air protection policy is then reviewed. Current policy limitations are also explored by applying policy network theory. Finally, air protection policy recommendations are proposed in consideration of the domestic situation.

## 2. Theoretical Framework and Methodology

A policy network comprises several actors, each having specific interest in a certain policy arena [35,36,37]. As an approach to determining the contributions of such actors in policy implementation, policy network theory has received increased attention in policy analysis [32,38,39,40]. A policy often involves many actors. If we take an environmental protection policy for example, it may involve many departments in governments, state-owned enterprises, energy firms and many others. Policy network theory defines policy-making as a process, in which many actors corporately make decisions and the implementation depends on the interaction among all of them [41]. The underestimation of interdependencies among these actors is bound to lead to disappointment [33,34]. The approach thus provides an effective method for gaining policy insight and evaluating outcomes [42]. In previous studies, some political scientists have argued that policy network theory is inadequate for explaining policy change [43,44] or better at explaining policy stability [45]. It is because this theory was thought to be an approach to classify the relationship between social groups and government constantly. And the network was thought to be fixed when the groups’ interest is met after changing information with government. The theory was accordingly thought to be better at explaining government’s certain policy action or policy stability than policy change [43]. However, other scholars have taken the opposite view. They insist the implementation and effects of a policy can be determined by analyzing resource exchanges among relevant actors after determining the network structure [46,47,48,49,50,51]. In reference [51], Compston redefined the policy network theory in view of this resource exchange so that the development of an existing policy can be thoroughly explained by taking resource exchange into account.

**Figure 2:**
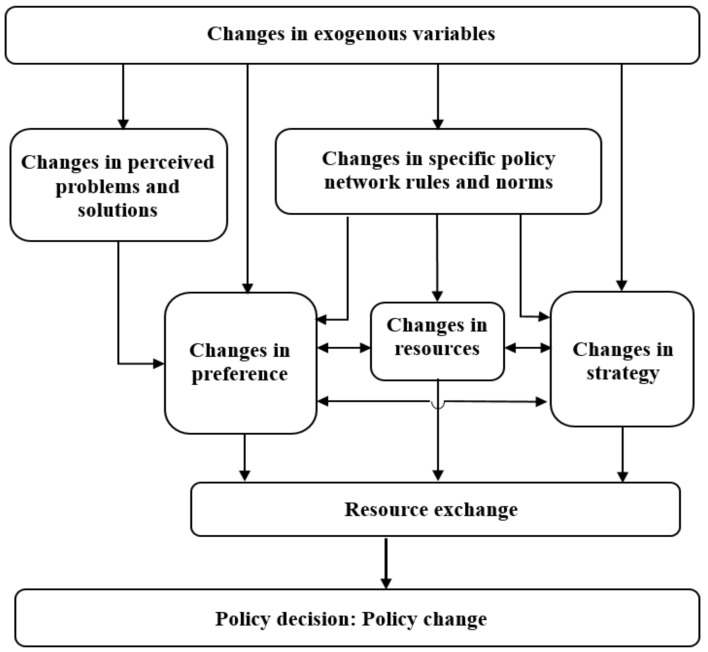
Policy network model explaining policy change (Source: Adapted from reference [52]).

Compston used the following six propositions that must be presumed to define policy network theory [51]. First, there are policy decisions. This proposition is a precondition for the other propositions. Second, there are policy actors (both individuals and groups) possessing tradable resources. Third, policy actors have respective policy preferences. Fourth, there are perceived policy problems and solutions. Fifth, based on their respective tradable resources, policy actors design strategies to maximize their chances of realizing respective policy preferences. Finally, there are incentives that regulate the behaviors of policy actors and the interactions among them. From the last five propositions, five crucial variables were extracted accordingly to explain policy changes: actors’ resources, actors’ preferences, perceived problems and solutions, strategies adopted by actors, and network-specific rules and norms. The way to explain policy’s change in a policy network is shown in Figure 2. However, the approaches to policy change are not uniform. By influencing resource exchange among policy network actors, changes in exogenous variables differentially lead to policy changes [51]. Based on this theory, Compston explained the development of climate policy in wealthy Annex I countries, which signed legally binding international agreements under the UNFCCC. Although not restrained by international agreements, Shyu confirmed that these exogenous variables also influenced policy changes in both non-developed and non-Annex I polities [52]. Compston’s theory has thus far been applicable across a variety of contexts [51]. However, it has not yet been applied to Chinese policy. This study therefore examined Shyu’s findings in the Chinese context [52].

Various scholars have adopted policy network theory to study policy changes in China’s cases, such as education [53], eco-city [54] and urban housing [55,56]. Besides, interdependency among actors in the course of policy changing and implementation can be explored by policy network theory [32]. In all of the researches, one point cannot be ignored is that China is both a developing and non-Annex I country in addition to being socialist. The entrepreneurial spirit of the Communist party has been praised for [57], while nepotism and bureaucratic corruption has been criticized [58]. As Marsh and Rhodes pointed out, political authority and political economy played key roles in establishing policy networks [59]. Thus, China’s socialist political system and unique economic structure should be considered as limiting factors in an analysis of resource exchange in the policy environment.

All Chinese documents, events, and statistics analyzed in this paper were collected from relevant policies, policy claims, regulations, and official reports published on government webpages and by mainstream media sources. To ensure the accuracy of analysis, all the selected data come from the national official database provided by National Bureau of Statistics. And the selected documents on air protection were frequently referenced in relevant studies. The interpretation of the data and the selection of documents rely on author’s judgement, which may result in controversy regarding the results. However, it should therefore be noted that this paper did not intend to explore correlations or causality among variables. Rather, the intent was to examine the effects of exogenous factors on policy networks. As such, international negotiations and agreements were analyzed as exogenous factors influencing Chinese domestic policy networks. This study applied policy network theory to verify the impact of exogenous factors on the development of Chinese policy networks by combining the reality of socialism with Chinese characteristics, and then explored the resource exchange limitations involved in Chinese policy changes.

This paper defined UNFCCC and its amendments as primary exogenous factors. UNFCCC’s ultimate objective is the prevention of dangerous anthropogenic interference in the climate system and to protect people from climatic danger caused by air pollution and extreme weather [60]. This study involved scientific analysis of the agreement and its supplementary protocol as exogenous factors influencing the Chinese domestic policy network. Compston’s model was therefore applied to the Chinese policy environment [51].

## 3. Status of Energy and Pollutant Emissions in China

China is a developing country undergoing rapid economic growth. However, such development is driven by low-income rates, relatively few worker benefits, high environmental costs, low social costs, a high rate of investment and exports, high consumption, and heavy contamination. China’s 2014 gross domestic product (GDP) was approximately $10.4 trillion, which represents about 13.43% of the global GDP. However, Chinese energy consumption was equivalent to 2972.1 million tonnes of oil in the same year, thus accounting for 23% of consumption worldwide. The unit “tonnes” (equivalent to 2240 pounds) was used here to be consistent with the unit used in the source data. This makes China the leading energy consumer [61]. China’s primary energy efficiency is therefore much lower than the world average. In addition, fossil fuels account for nearly 90% of national primary energy consumption although the resulting pollution is the leading environmental threat to public health [62]. Primary energy efficiency and the energy consumption structure are the main reasons China has had little control over such pollution.

### 3.1. Energy Status

Chinese government data indicate that total energy consumption was equivalent to 4260 million tonnes of coal in 2014, which increased by 2.6% from 2013 [63]. These are the same statistics produced by British Petroleum [61]. Fossil fuels are the main sources of energy in the China. Over the past 20 years, the greatest resources have been coal and petroleum, which result in large amounts of pollution, including nitrogen oxides, sulfides, and soot particles (Table 1). Although the Chinese electric industry has rapidly developed, coal and petroleum accounted for over 83% of energy usage in 2014.

### 3.2. Waste Gas Emissions Status

**Figure 3:**
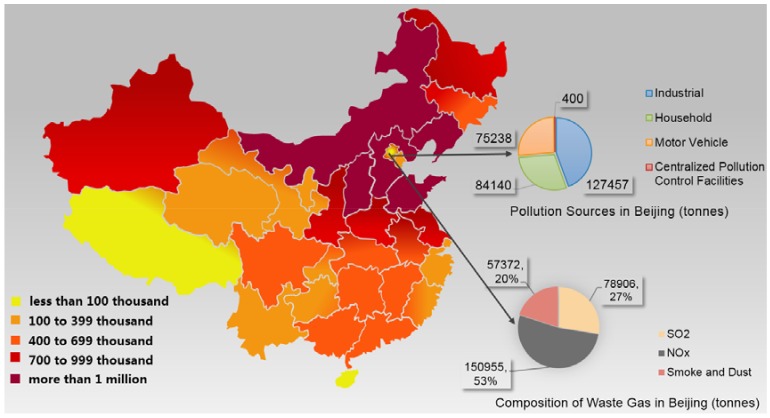
Waste gas emissions in different districts (tonnes) (Source: Drafted by authors according to online data from National Bureau of Statistics [64]).

Chinese overreliance on fossil fuels has led to a large amount of waste gas emissions, including sulfur dioxides, nitrogen oxides, and smoke and dust. These three pollutants have been monitored by the Chinese Ministry of Environmental Protection (MEP) since 2011. Waste gas emission numbers for 2015 in all districts are available in Figure 3. It is evident that air pollution is most serious in northeastern China.

**Figure 4:**
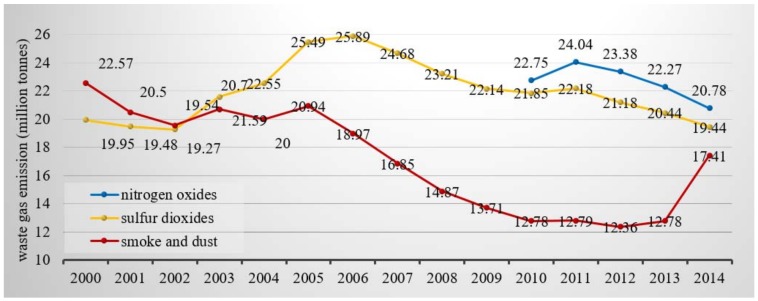
Variations in China’s waste gas emissions (Source: Drafted by authors according to National Environment Statistical Bulletin 2000–2014 [65]).

China signed the Kyoto Protocol in 1998 and promised to further reduce emissions during a second commitment period in 2013. Sulfur dioxide emissions have been effectively curbed over the last 10 years. Having been monitored since 2010, nitrogen oxide emissions have also decreased. However, smoke and dust emissions increased dramatically, moving up 35% from 2010 to 2014, and the portion of total waste gases rose by 8% during this time (Figure 4) [65]. Smoke and dust emissions especially increased in 2014, going up more than 30% (Table 2), while sulfur dioxides and nitrogen oxides fell nearly 7% each year. Although municipal runoff significantly increased in 2014, industrial emissions were the main causes of pollution. Although China was one of the earliest parties to enter the UNFCCC, the nation only pledged to undertake “common but differentiated responsibilities” as a developing country until 2010. In 2010, the Chinese government made a commitment to the international community to lower its carbon dioxide emissions per unit of GDP and increase its share of non-fossil fuels in primary energy consumption.

## 4. Effects of Exogenous Variables on Chinese Governmental Air Protection Policy

China’s response to international climate agreements and negotiations has involved four developmental stages, as follows: (1) UNFCCC adoption (1992 to 2005); (2) Kyoto Protocol (2006 to 2012); (3) Kyoto Protocol (2013 to 2020); and (4) a new course toward 2020; in this period, a new global agreement may be negotiated to replace the Kyoto Protocol. This section discusses the first three stages.

### 4.1. UNFCCC Adoption (1992 to 2005)

The UNFCCC was first opened for signature during the United Nations Conference on Environment and Development (UNCED) in Rio de Janeiro in 1992. The Chinese central government signed this convention and promised to take on “common but differentiated responsibilities” [60]. The Kyoto Protocol was developed as a supplementary provision of the UNFCCC to explicitly stipulate an obligation on the part of developed countries to limit greenhouse gas emissions during UNFCCC COP 3 (i.e., the third session of the Conference of the Parties) in 1997. China submitted its approval of this convention to the Secretary-General of the United Nations in 2002. The legally binding Kyoto Protocol became effective during UNFCCC COP 11 in February 2005.

China’s initial response to the Kyoto Protocol was forming the Sustainable Development Research Centre at the Chinese Academy of Social Science in 1997. This organization primarily focused on academic and policy research in the field of sustainable development from a social economy perspective. Nevertheless, China disregarded many of its obligations to maintain massive exports and low “China prices” [66]. Thus, resource input and consumption remained very high. This continued until 2003, when the Communist Party of China proposed the Scientific Outlook on Development. This was done to mitigate the imbalances and unsustainability in Chinese development. China established its first target for energy conservation and emission reductions with its Eleventh Five-Year-Plan in 2005, which proposed cutting energy intensity per unit of GDP by 20% from 2006 to 2010 [67]. By legally referring to Paragraph 1, Article 4 of the UNFCCC, this action made China the first Annex II country to confront climate change. This indicated a transition in which the government began to regard air quality protection as not only a public problem but also a policy problem.

### 4.2. Kyoto Protocol Commitment (2006 to 2012)

Formal UNFCCC negotiations on waste gas reduction targets subsequent to 2012 were long and difficult [52]. In 2007, the Bali Road Map was passed and became effective during COP 13, which was held in Bali, Indonesia. This measure introduced dual-track negotiations to involve both developing and developed countries that did not sign the Kyoto Protocol, including the United States. It was designed to reach an agreement on new arrangements for the post-Kyoto period (2012–2020) at UNFCCC COP 15 in Copenhagen two years later. However, an agreement was not reached. Later, the Durban Conference (i.e., UNFCCC COP 17) adopted the Durban Package Outcome and established the “Durban Platform”, which created a process by which all parties to the United Nations Climate Change Conference (i.e., UNCCC) would commit to a new and legally binding agreement to reduce waste gas emissions [68]. This agreement will become effective in 2020. The second Kyoto Protocol thus identified 2020 as the expiration date for the first commitment period. The conference maintained the validity of both the Kyoto Protocol and the UNFCCC, therefore completing the Bali Road Map.

After the first voluntary reduction program was proposed by the Eleventh Five-Year-Plan, a basic plan for building a harmonious socialist society was stated in the Sixth Plenum of the Sixteenth Party Central Committee in 2006. This is when China prioritized environmental protection on its national economic development agenda. Until Annex II countries were required to submit nationally appropriate mitigation actions (NAMAs) during UNFCCC COP13 in 2007, other developing countries had begun to develop their own emission reduction measures. At that time, China established the Specialist Committee of National Climate Change (also known as the Chinese Climate Change Think-Tank) and the National Leading Committee on Climate Change, which was headed by the prime minister and included the heads of more than 20 departments (e.g., the Ministry of Foreign Affairs, Ministry of Land and Resources, and the National Development and Reform Commission). China’s National Climate Change Program was adopted to identify waste gas emission reduction targets, main focus areas, basic principles, and relevant policy measures [69]. The Chinese government publicized its emission reduction targets just before COP15 in Copenhagen. In 2009, China worked for a practical solution to establish a green, low-carbon, and sustainable development path by proposing the Decision on Active Response to Climate Change, which was passed by the Standing Committee of National People’s Congress (NPC, the highest organ of state authority) [70]. The concept of a low-carbon economy was then written into the Premier’s Report on Government Progress and the 12th Five-Year-Plan [71], which established the guidelines for national economic and social development from 2011 to 2015.

### 4.3. Kyoto Protocol Commitment (2013 to 2020)

In 2014, China signed the China-US Joint Statement on Climate Change, thereby committing to reaching a CO_2_ emissions peak and increasing the proportion of non-fossil fuels in its primary energy consumption to around 20% by 2030. At COP21 in Paris in November 2015, the Chinese government submitted its intended nationally determined contributions to the UNFCCC Secretariat, thus proposing to cut its 2005 CO_2_ emissions per unit of GDP by 60–65% in 2030. Regarded as the third landmark international legal document following the UNFCCC and the Kyoto Protocol, the Paris Agreement was also approved at this conference. The agreement required all parties to take more emission reduction efforts than those associated with the intended nationally determined contributions in addition to establishing a new mechanism of global stocktaking to mitigate pollution emissions and fund commitments on a five-year cycle starting in 2023.

**Figure 5:**
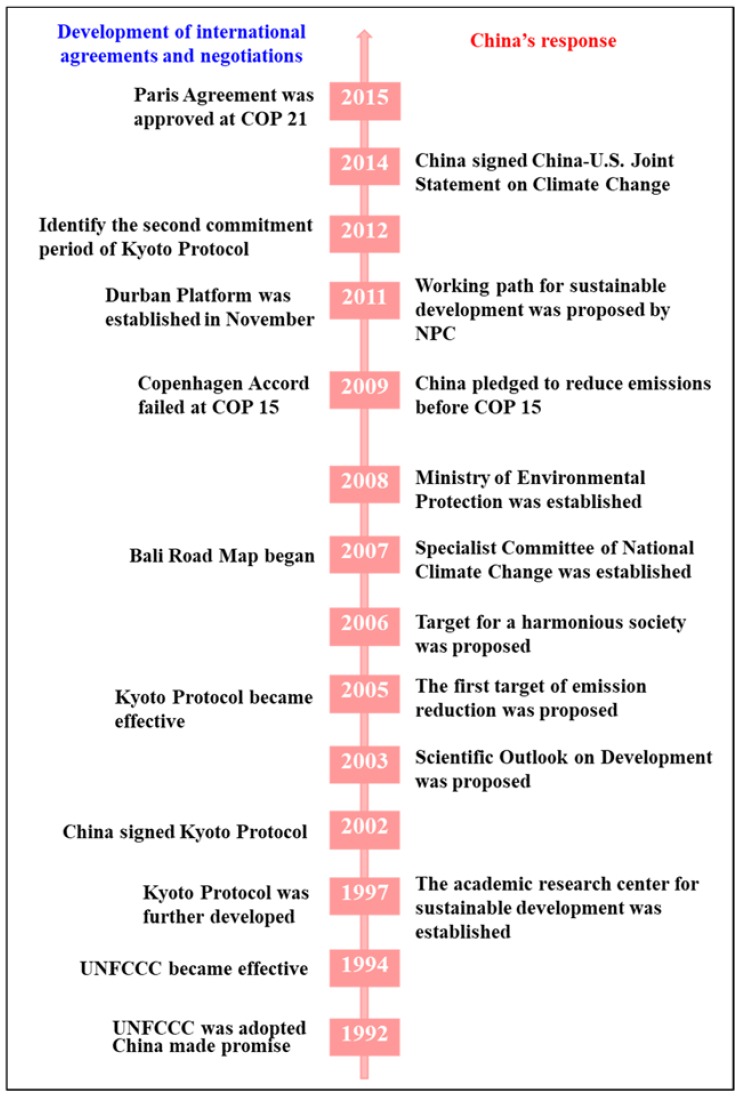
China’s policy response to international agreements and negotiations (Source: Compiled by authors).

China’s actions during international negotiations involving climate change since 1992 are shown in Figure 5. There is little evidence to demonstrate how exogenous factors (e.g., the UNFCCC and Bali Road Map) may have significantly affected changes in Chinese policy. Rather than the unilateral effects found in studies on other countries, Chinese policy change seems to have resulted from interactions between the Chinese government and the international community. To some extent, international agreements and negotiations may be driven by China’s actions.

## 5. Analysis of Air Protection Policy Changes

This section first reviews Chinese governmental air protection policy development. Policy network theory is then applied to analyze policy changes and resource exchange limitations.

### 5.1. Analysis of the Air Protection Policy Development Process

This section is divided into three chronological stages (i.e., prior to 1990, during the 1990s, and after 2000) summarizing policy characteristics.

#### 5.1.1. Prior to 1990: Administrative Control

Chinese air pollution control policies date back to the 1956 *Hygienic Standards for the Design of Industrial Enterprises*. This rule defined 34 hazardous atmospheric substances in residential quarters and a maximum permissible concentration of 120 hazardous airborne substances in the workshop environment. In 1973, the State Development Planning Commission (SDPC, renamed the National Development and Reform Commission in 1988) issued proposals to further chimney dedusting work; the content focused on boiler improvements to eliminate soot. The *Trial Standard of Industrial Three Wastes Discharge* was later enacted as China’s first environmental standard by the SDPC, the State Infrastructure Commission (SIC, formerly the Ministry of Housing and Urban-Rural Development), and the Ministry of Health (MH, formerly the National Health and Family Planning Commission). This agreement prescribed industrial pollution emission standards. Control over environmental pollution was first signed into law in 1979 under the *Environmental Protection Law of the P.R. China (for Trial Implementation)*. This law included clauses on pollution standards, soot dedusting, production plants, and production processes.

After the “Reform and Opening” in 1978, Chinese society struggled to find a path to effective legal reform as a whole. This accelerated the promotion of laws to prevent and control air pollution. Thus, the *Law of the P.R. China on the Prevention and Control of Atmospheric Pollution* (the first atmospheric protection law) was enacted in 1987. This is the first instance in which China incorporated atmosphere protection into law. Moreover, China issued the *Ambient Air Quality Standard (GB 3095—1982)*, which was the first standard for atmospheric environmental quality, in 1982. State Council departments promulgated many successive regulations and standards, including the *Rules on Technology Policy of Prevention and Control of Coal-burning Pollution (1984)*, *Policing Method of Urban Smoke and Dust Control Area (1987)*, *Interim Procedures for Developing Coal Briquette for Civilian Use (1987)*, *Standard of Smoke and Dust Emission for Boiler (1983)*, and the first batch of automotive exhaust emission standards. Chinese local governments took many measures to curb heavily polluting enterprises during this period, such as requiring them to manufacture other products, amalgamating them with other industries, suspending operations, and even closing facilities [66].

According to above policies retrospective, primary air protection measures were unilaterally operated by the government. Administrative power was used to control air pollution, and industrial firms were targeted as major sources of pollution [72]. New rules and laws were issued in conjunction with growing industrial energy demands. Chinese management standards for ambient air quality were nationally unified, and environmental air protection entered a new stage of law-based administration during this time. Boiler soot and automotive exhaust were also monitored as preventative measures. Further, increased government enforcement marked a time in which the Chinese administrative stance on atmospheric protection would shift to integrated control.

#### 5.1.2. During the 1990s: Integrated Control

China increasingly took measures to curb pollution during this time [73,74], as follows:

**Introducing a market mechanism into ambient air management.** The State Environmental Protection Bureau (SEPB) selected six pilot cities in which quantities discharged from pollution sources could be traded with other sources if the totals did not exceed the maximum allowed. The government began to levy charges on SO_2_ emissions by adjusting the leaded petrol consumption tax. Meanwhile, bidding was carried out in pilot cities to introduce competition into the environmental assessment system. The SEPB also released the *Principle and Method of Determining Emission Indicators of DP* and the *Administrative Measures of Discharge Permit (DP) (Frame Draft)*, which formed the guidelines for the pollutant discharge permit system. Thus, no individuals or companies were allowed to discharge pollutants into the environment until receiving discharge permits (DP), which were issued by the provincial environmental protection bureaus. The *National Programme for Environmental Protection (1993–1998)* was then established to strengthen DP management.

**Improved laws on air pollution prevention and control.** The Supervision and Administration of Automobile Exhaust Pollution was issued in 1990. The Enforcement Regulations for Law on Prevention of Air Pollution of P.R. China was also introduced to control waste gas emission concentrations while actively monitoring the amounts. The laws were revised to reduce the impact of lampblack in residential environments while adding regulations to prevent acid rain, control SO_2_ discharge, and improve the environmental protection management of catering services. In addition, the first revision of the Ambient Air Quality Standard (GB 3095—1996) was enacted in 1996. This revision adjusted pollutant test standards, regulated methods for sampling and analyzing ambient air, and defined the validity of statistical data.

**Improved supporting policies.** The *Decision of the State Council on Several Issues Concerning Environmental Protection* was released at the fourth National Environmental Protection Conference in 1996. The decision ordered main polluting sources formerly regulated by regional organizations (e.g., industrial and residential areas) to achieve respective national environmental standards. In 1998, the State Environmental Protection Administration (SEPA, formerly the SEPB) distributed the *Outline of China Environmental Protection Work (1998–2002)* to establish environmental protection targets and proclaim that severely polluting factories would be closed down if they did not adjust to China’s new industrial structure within a certain time period.

The above measures indicate that administrative control of the central government’s environmental protection sector had been elevated to ministerial status. Policy instruments tended to be more comprehensive compared with the previous period. For instance, the environmental impact assessment system played a preliminary prevention role, while the DP system controlled discharge amounts midway through production, and the system undertaking treatment within a prescribed time period performed disposal functions at the final stage. During the 1990s, government control of various pollution sources was integrated into a single preventative measure focusing on industrial discharge.

#### 5.1.3. After 2000: Cooperation and Common-Governing

With China’s accelerating industrialization, urbanization, and regional economic integration, atmospheric pollution has become a multi-agent and more comprehensive issue. Many policies and laws on air pollution control were successively released, as follows:

**Launching joint countermeasures against atmospheric pollution.** During the 2008 Olympics, six provinces and municipalities, including Beijing and Tianjin, signed the *Beijing Air Quality Assurance Measures in the Twenty-ninth Olympic Games*. These measures primarily aimed to prevent pollution from raised dust, vehicular exhaust, industrial emissions, and coal burning [75]. In 2010, the MEP (formerly the SEPA), the National Development and Reform Commission (NDRC), and seven other ministries jointly developed guidelines on the prevention and control of air pollution to improve regional air quality. These established mechanisms to jointly prevent atmospheric pollution that would become effective by 2015. The “*Twelfth Five-Year Plan*” *on Air Pollution Prevention and Control in Key Regions* was China’s first comprehensive air pollution control program [76]. It was jointly approved by the MEP, NDRC, and the Ministry of Finance. The State Council then announced the *Air Pollution Prevention and Control Action Plan*, which was considered the strictest environmental action plan at the time. In 2014, the MEP established goals for air prevention and control responsibility in 31 provinces (i.e., autonomous regions and municipalities) to explicitly define different regional priorities and tasks.

**The promotion of informational freedom and public participation**. Media outlets and environmental volunteers had occasionally been participating in air quality activities since October 2011, including “The Campaign for Measuring Our Motherland’s Air Quality” and “Give Advice to MEP”; many of these events were designed to force authorities to take relevant measures [77]. As a result, the MEP revamped its *Ambient Air Quality Standard (GB 3095—2012)* to include PM_2.5_ as a testing metric. This new standard also added limits for the concentration of ozone (O_3_) and lowered the limits on nitrogen dioxide (NO_2_) and PM_10_. Amendments to the *Environmental Protection Law of the P.R.C.* were then passed by the NPC Standing Committee in 2014. This included a chapter on open information to highlight improvements to laws on public participation, public interest litigation, and democratic supervision [78].

**Adding air protection to governmental examination standards**. Obligatory sulfur dioxide (SO_2_) emission reductions were included in the national economic and social development plan. According to the *Comprehensive Work Plan for Conserving Energy and Reducing Emissions for the Twelfth Five-Year Plan Period*, these reduction targets were integrated into the official scope of assessment for 2011 [76]. The fourth revision to the *Law on Prevention and Treatment of Air Pollution (2015)* stipulated that local government heads would be interviewed if periodic waste gas emission reduction targets were not met.

**Continuously refining the legal system.***The PR China Cleaner Production Promotion Law* was promulgated in 2003 to reduce pollution across all production processes; it was revised in 2012. In the revision, the Chinese government specified increased penalties for illegal companies and established a planning system to promote cleaner production [79].

According to the above retrospective and conclusion of relevant policies, the administrative status of the central government’s environmental protection sector was further increased to the Organs Composing the State Council during this time. Air pollution prevention was then included in a contingency plan for environmental emergencies, and relevant policies were frequently introduced to combat increasing urban haze pollution. Policy dynamics concentrated at the top of local governments to guide intersectoral collaboration, cross-regional cooperation, and involve societal participation.

### 5.2. Analyzing Policy Actors within the Policy Network

**Figure 6:**
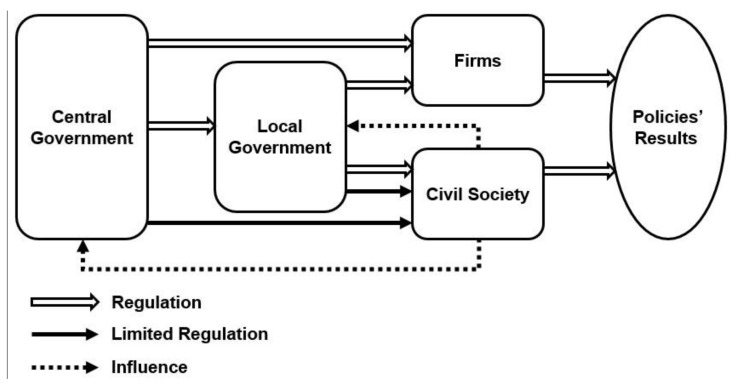
Chinese air pollution prevention and control policy network (Source: Drafted by authors).

Policy problems are derived from the interaction between triggering devices and initiators [80]. Compared with international agreements, internal events are the more important triggering devices. The problems of Chinese air pollution control have worsened with increasing domestic complaints. Although public opinion can influence policymakers to confront the issue to some extent, the initiator is still clearly the central government in Chinese political system. The policymakers in the central government evaluate and select domestic events, then upgrade it into a policy problem. The development of air protection policy is accordingly started with the policy problem being put on the government’s policy agenda [52]. Therefore, the central government is the primary policy actor and formulates a corresponding top-down policy network, which contains the other actors (e.g., firms, local governments, and civil society) (Figure 6). Policies released by the Chinese central government are therefore immediately effective for local governments and other firms, including energy enterprises and factories discharging air pollutants. Because Chinese living conditions are regionally variable, the central government has not issued a great deal of regulations or rules directed at civil society. Instead, local governments release practical policies according to provincial characteristics by following central policy guidelines. Finally, policy results are displayed by both firms and civil society.

#### 5.2.1. Central Government

In the Chinese political structure, the central and local governments are vested with unbalanced political status and administrative responsibility, thus resulting in different values and preferences according to hierarchical grade [39]. The central government is the highest authority. It reserves the power to make decisions and issue orders. As head policymaker, the central government must consider both efficiency and fairness when operating macroscopically. Its sustainable development proposition was intended to balance the environmental burden while developing the economy. The State Council is a key actor in the central government. However, it is composed of many ministries and commissions, each of which play important background roles in the policymaking process as independent actors. They can be divided into three groups according to their influence: the most influential policy actors; other influential policy actors; and other involved policy actors. Role, preferences and strategy of these actors are shown in the following tables (Table 3, Table 4 and Table 5). These entities are more or less equal. As such, laws and regulations are the results of collective bargaining. For example, Article 54 of The Law of the PR China on the Prevention and Control of Atmospheric Pollution was never used because the administrative law-enforcement subject was indeterminate. An MEP officer attributed this to significant opposition upon introduction in an interview [81]. The subject of law enforcement was deemphasized to prevent this issue from blocking the article’s entire revised edition.

#### 5.2.2. Local Government

Local Chinese government plays a political role in executing decisions and policies issued by the central government. Progress towards these goals depends on local government enforcement. However, the central government is driven by different values. Local government must therefore play an increased economic role by paying more attention to GDP growth than environmental protection to achieve desirable economic development data and political promotion. When the local economy is heavily reliant on industrial enterprise, the government will order the local Environmental Protection Bureau to refrain from examination to prevent economic burdens caused by punishments that result in increased unemployment. As such, the Chinese MEP has been called one of the most awkward departments in the world [83].

#### 5.2.3. Relevant Firms

Two types of actors belong to policy network firms relevant to this study. One includes centrally administered and state-owned energy enterprises (e.g., the China National Petroleum Corporation (CNPC) and the China Petroleum & Chemical Corporation [Sinopec Corp.]). The other involves any company listed as a heavy polluter.

State-sanctioned energy enterprises constitute a monopoly in petroleum processing and oil refining because of the associated strategic value [84]. This has resulted in a closed and noncompetitive market. Such companies thus received significant criticism from civil society as a result of heavy smog. Sinopec’s chief engineer confronted public outcry by arguing that oil quality conformed to state standards and that energy companies had no right to influence these standards by overstepping regulations set by the petrochemical standard committee (i.e., the National Technical Committee of Petroleum Products and Lubricants, NTCPPL). However, 24 of the 43 NTCPPL committee members were officials or employees of these petrochemical companies at the time [85]. Chinese fuel quality standards have therefore been dominated by state-owned petrochemical companies rather than a neutral agency. This may put the integrity of the committee into question.

Companies listed as heavily polluters are also influential actors. Local governments tend to confer protection to these companies due to concerns about the economic achievement record, although this protection may not always be necessary. The record influences local government officials to give polluting companies preferential policy treatment or financial support instead of subjecting them to supervision from local environmental departments in order to meet implicit economic quotas. Some of these companies therefore become “zombie enterprises”, meaning they require assistance to continue operating. For example, Bao Steel Co. Limited obtained almost $0.26 billion in subsidies (according to the exchange rate on December 31, 2015) while listing a $0.2 billion net loss during the first three quarters of the 2015 fiscal year. This type of support is not unusual among Chinese local governments. However, research indicates that government fiscal subsidies inflated the earnings of listed steel companies, thus influencing profitability that did not otherwise meet expectations [86].

#### 5.2.4. Civil Society

China contained 731 million netizens (i.e., Internet users) as of December 2016. Among them, 695 million used mobile phones to access the Internet [87]. Astounding social media advances (e.g., Weibo and WeChat) have greatly involved Chinese civil society in discussions about social phenomena and government action. Many researchers agree that the Internet has positively influenced public politics and increased public participation in government decision-making [88,89]. For example, when official air test results received increased criticism from mainstream Internet media sources, the MEP promptly responded to avoid public outcry [90]. It is clear that civil society has become increasingly involved in the policy network as a serious actor.

## 6. Discussion and Policy Recommendations

Chinese air protection policy developments are the result of resource exchange among policy network actors. Little empirical evidence has demonstrated that international agreements and negotiations have highly influenced Chinese air protection policy. Rather, such policy development has primarily been influenced by changes in civil needs and domestic public opinion.

One reason for this may be the lack of a legally binding commitment on gas emission reductions. Although the Chinese government has signed several international agreements, most were not legally binding. China has always stressed that it should not share equal responsibility as developed countries, which industrialized a century earlier. Compared to exogenous factors, domestic complaint and public opinion are the main influences behind government action to improve air quality. However, a strong desire for economic growth has resulted in severe fog and haze. This has resulted in increased regulations, policies, and laws.

China has developed laws and policies based on voluntary mechanisms rather than enforcement. Lacking a legal framework, domestic policy actors primarily take action based on perceived benefits and preferences. Local governments are likely to prefer increased revenue and GDP when deliberating over economic growth and environmental protection. Environmental protection departments tend to become ineffective while firms tend to pursue higher profits and ignore environmental payments. In addition, momentary doubts about environmental improvement measures can halt regulations and policies from achieving effective outcomes.

1. Based on previous analysis on the characteristic of policy network and relevant actors, this study suggests the following five policy recommendations from the aspect of a bureaucratic system, an energy market running mechanism, and others. The target is overcoming the associated limitations and obstacles during the process of policy-making and implementation. Raise the effectiveness of policy network rules and norms

To make sure a policy can be implemented as planned, relevant rules and norms must be clear and effective. Therefore, policymakers should fully consider the strategies and preferences of network policy actors. It is not sufficient to simply take policy destination into account during policymaking. Rather, a policy should become a common achievement through coordination and help from all actors. An actor’s strategy will therefore adjust to the preferences of other actors. Policy outcome is therefore a collective strategy. If the effective rules or legal binding power to regulate independent behavior are missing, expected policy outcomes are difficult to achieve. This approach is key to raising the effectiveness of policies and improving outcomes.

2. Increase the Power of Supervisory Departments

The Chinese bureaucratic system should be increasingly supervised. The MEP should have played a significant role in the current policymaking network by superintending and penalizing. As the previous analysis on local government shows, however, the environmental protection departments at locals do not receive the attention they deserve. They are in the same administrative hierarchy as the developmental arms of local governments. Although the environmental protection offices in the central government were elevated to “super ministries” in 1974, the MEP was still treated as an insignificant department. Due to the absence of a higher executive power, local environmental supervision departments are not able to effectively curb incompatible behaviors or violations by peer departments. In summary, endowing environmental supervision offices with increased power is important to guarantee policies implementation and obtain desired results.

3. Breaking the Market Monopoly

Energy firms is another crucial actor in the network of Chinese air protection policies. Because waste gas emissions are clearly influenced by fossil fuel energy usage. However, improving the quality of fossil fuels resulted in bureaucratic gamesmanship between the government and the energy enterprises. For example, it is difficult for the government and oil companies to agree on the price of upgraded oil due to cost differences. The petroleum industry must be run by state-owned monopolies because petroleum security was defined as the core of state energy security by the Chinese supreme leader [91]. This monopoly poses difficulty in improving the quality of oil and adjusting the prices of oil products. The government is thus forced to maintain current quality at a tolerable price. To improve air quality, the power of state-owned energy companies should be diminished by restoring market competition by encouraging non state-owned enterprises and international capital to flow into the energy market.

4. Deepening Energy System Reform

China’s current energy system involves many policy actors. It includes the state-owned energy firms and the national and local branches of the National Development and Reform Commission. It will be impacted whether the energy market is regulated or renewable energy is developed. The resulting resource exchange among policy network actors will inevitably change the interests of some actors, especially those within the current energy system. To lower the associated barriers, the central government must standardize the energy market and advance laws to facilitate energy system reform. For example, the NTCPPL and other national technical committees of energy products should consist of neutral and impartial professionals. In addition, the Energy Administration cultivates massive corruption. Many government dignitaries and top managers of state-owned enterprises have been unseated since the Eighteenth National Congress of the Communist Party of China. Corruption was common in all state-owned petroleum, power, and coal enterprises in addition to national and local branches of the National Development and Reform Commission. It is therefore necessary to deepen energy system reform in terms of improving the system and against corruption for gaining expected policy results.

5. Redefine the Assessment System for Official Achievement

Given the performance and strategy of local governments mentioned above, the assessment system for official achievement should be designed environmentally friendly. By increasing the index weight for environmental protection in the assessment system, it may urge local government officials to pay more attention to the environment. The index weight should be strong enough to influence local officials to support air quality improvements. Further, a redefined assessment system should be oriented to the careers of these officials by including a formal accountability mechanism to ensure that precautions are taken and supervision is practiced. That is, officials will face declined promotions and even worse consequences if their achievements on environmental protection do not meet standards during their tenure. Local governments will then pay increased attention to air quality at the expense of GDP. The assessment system should also be designed according to indigenous features to address extreme developmental imbalances in the different regions of China.

## 7. Conclusions

This study examined Chinese air protection policy using Compston’s policy network theory. Results indicated that internal factors (e.g., policy actor preferences) were the main influences behind changes in Chinese air protection policy. As a socialist country with a developing economy and the largest population worldwide, little evidence was found to demonstrate that exogenous factors had influence over China’s policies. To some extent, China played a key role in urging the need for international agreements, including those on air quality protection. This paper comprehensively explored the reason policy outcomes are always below expectations by analyzing the characteristics of policy actors in the context of China’s political background. The lack of a legal enforcement framework decreases the effectiveness of current policy. Problems in the energy and bureaucratic systems impede air protection policy and breed corruption. To address these concerns, the central government should establish a sound legal framework to enforce and deepen relevant system reform based on sufficient study of the strategies played by other policy network actors.

## Figures and Tables

**Table 1:** Energy consumption structure of China ^1^.

Type of Energy	1994		2004		2014	
	Quantity (10^4^ tec)	Percentage (%)	Quantity (10^4^ tec)	Percentage (%)	Quantity (10^4^ tec)	Percentage (%)
Coal	92,052.75	75.0	161,657.26	70.2	281,160	66.0
Petroleum	21,356.24	17.4	45,825.92	19.9	72,846	17.1
Natural gas	2332	1.9	5296.46	2.3	24,282	5.7
Primary Energy	6996.01	5.7	17,501.36	7.4	47,712	11.2

^1^ Source: Adapted from British Petroleum Statistical Review [61].

**Table 2:** Emission structure of smoke and dust ^1^.

Type of Source	2013		2014	
	Quantity (Million Tonnes)	Annual Pace (%)	Quantity (Million Tonnes)	Annual Pace (%)
Industrial Emission	10.95	6.4	14.56	32.3
Municipal Runoff	1.24	−13.3	2.27	83.3
Vehicle Exhaust	0.59	−4.8	0.57	−3.4
Total Emission	12.78	3.6	17.41	30.2

^1^ Source: National. Environment Statistical Bulletin 2013, 2014 [65].

**Table 3:** The most influential policy actors in central government.

Actor	Role	Preferences	Strategy
State Council’s Development Research Centre	Offer scientific analysis and policy suggestion	Promote economic growth and maintain social stability	Promote its own reports to facilitate the debate in a new direction [39]
National Development and Reform Commission	Guide macroeconomic controls on the overall economic system reform	Achieve economic sustainable growth and social sustainable development.	Intervene at fuel prices, draft and coordinating implementation relevant policies.
Ministry of Environmental Protection	Establish sound basic system of environmental protection	Formulate and organize the implementation of laws and regulations, supervise and coordinate major environment problems by exercising enforcement power.	Make environmental impact assessment, monitor environment, release comprehensive report and major information.

**Table 4:** Other influential policy actors in central government.

Actor	Role	Preferences	Strategy
Ministry of Finance	Make fiscal policies to promote the protection of ecosystem.	Promote value for money for the industry of environmental protection by rolling back spending.	Promote the legislation of Environmental Protection Tax Law and set up special funds.
Ministry of Transport	Make transport planning and management to reduce the pollutant emission from traffic and transportation system.	Transportation facilities are the last reasons which cause air pollution issue while the system is sufficient to meet the demand of economic development.	Make relevant regulations on the administration of the transportation sector environmental protection.
Ministry of Housing and Urban-Rural Development	Held responsible for promoting building energy conservation and urban emission reduction.	Current system of housing construction standard and urban-rural development could not increase pressure for environmental protection.	Be silent when environmental issues came and only recognized heating system was the final straw that broke air’s back [82].

**Table 5:** Other involved policy actors in central government.

Actor	Role	Preferences	Strategy
Ministry of Science and Technology	Propose policies and measures to promote the commercialization of research findings for the prevention of air pollution.	Current institution and macroscopic strategy of scientific progress could support domestic economic sustainable development.	Pick over and popularize relevant achievements and applications in scientific research.
Ministry of Industry and Information Technology	Supports and gives guidance to raise industrial energy efficiency and to advance environmental industry.	Upgrading the industrial structure smoothly to embark on a new path of industrialization.	Make cleaner production assessment index system for certain industries or departments.
Ministry of Commerce	To regularize industrial practices and corporate behavior towards green recycle.	Keep economic and market trade advancing without the obligation to clean air.	Supervise relevant departments to change, only if these could not impact economic and trade.
